# Comparison of accelerated partial breast irradiation via multicatheter interstitial brachytherapy versus whole breast radiation

**DOI:** 10.1186/1748-717X-7-53

**Published:** 2012-03-29

**Authors:** Daniel J Ferraro, Adam A Garsa, Todd A DeWees, Julie A Margenthaler, Michael Naughton, Rebecca Aft, William E Gillanders, Timothy Eberlein, Melissa A Matesa, Imran Zoberi

**Affiliations:** 1 Department of Radiation Oncology and Siteman Cancer Center, Washington University School of Medicine, Saint Louis, MO 63110, USA; 2 Department of Surgery and Siteman Cancer Center, Washington University School of Medicine, Saint Louis, MO 63110, USA; 3 Department of Medicine and Siteman Cancer Center, Washington University School of, Saint Louis, MO 63110, USA; 4 Department of Surgery, John Cochran Veterans Hospital, Saint Louis, MO 63106, USA; 5 Department of Radiation Oncology, 4921 Parkview Place - LL/Campus Box 8224, Saint Louis, MO 63110, USA

## Abstract

**Background:**

Brachytherapy as adjuvant treatment for early-stage breast cancer has become widely available and offers patients an expedited treatment schedule. Given this, many women are electing to undergo brachytherapy in lieu of standard fractionation radiotherapy. We compare outcomes between patients treated with accelerated partial breast irradiation (APBI) via multicatheter interstitial brachytherapy versus patients who were also eligible for and offered APBI but who chose whole breast radiation (WBI).

**Methods:**

Patients treated from December 2002 through May 2007 were reviewed. Selection criteria included patients with pTis-T2N0 disease, ≤ 3 cm unifocal tumors, and negative margins who underwent breast conservation surgery. Local control (LC), cause-specific (CSS) and overall survival (OS) were analyzed.

**Results:**

202 patients were identified in the APBI cohort and 94 patients in the WBI cohort. Median follow-up for both groups exceeded 60 months. LC was 97.0% for the APBI cohort and 96.2% for the WBI cohort at 5 years (ns). Classification by 2010 ASTRO APBI consensus statement categories did not predict worse outcomes.

**Conclusion:**

APBI via multicatheter interstitial brachytherapy provides similar local failure rates compared to WBI at 5 years for properly selected patients. Excellent results were seen despite the high fraction of younger patients (< 60 years old) and patients with DCIS.

## Introduction

Prospective randomized controlled trials have established breast conservation therapy (BCT), consisting of partial mastectomy and adjuvant radiation therapy, offers equivalent disease control in women with Stage I and II breast cancer as compared to mastectomy and significantly superior disease control when compared to partial mastectomy alone [1,2]. In the setting of ductal carcinoma *in situ*, adjuvant radiation therapy has been shown to increase local control [3-5].

The standard method for administering breast irradiation as a part of BCT is whole breast irradiation (WBI) delivered in five daily fractions per week for several weeks. In an effort to expedite radiation therapy, accelerated partial breast irradiation (APBI) techniques have been developed. Early studies of APBI have described impressively low ipsilateral breast tumor recurrence (IBTR) rates [6,7]. We have offered APBI or WBI therapy as treatment options for early-stage breast cancer in appropriately selected patients since December 2002. The purpose of this report is to review the outcomes of our multicatheter APBI in comparison to a cohort of patients eligible for and offered APBI but treated with WBI during the same time interval.

## Methods

### Patient selection and data analysis

Patients diagnosed with T_is_-T_2 _N_0 _M_0 _(AJCC 6^th ^Edition) unifocal breast cancers ≤ 3 cm in size from December 2002 through May 2007 who underwent breast conserving therapy with negative margins and received adjuvant radiation therapy with either APBI using multicatheter interstitial breast irradiation or WBI via external beam irradiation were identified. Patients who received neoadjuvant systemic therapy were not eligible for APBI and are not included in this review. All patients included in the study were eligible for and offered the option of APBI or WBI at initial radiation oncology consultation. Patients who were not eligible for APBI and/or only offered WBI were excluded from this study to limit bias. Three patients with synchronous primaries in bilateral breasts were identified in the APBI cohort with each breast cancer considered independently.

APBI was generally not recommended to patients younger than 40 years. However three patients in the APBI cohort were in their thirties at diagnosis. Two of these patients had prior radiation and APBI was offered to minimize the volume of reirradiation. The third patient declined WBI but was willing to do APBI. Given the presence of these three patients in the APBI cohort, we included patients seen in the study time period who were in their thirties, who received WBI, and who would have met the other eligibility criteria for APBI.

Breast cancer subtype for invasive cancers was approximated using estrogen receptor (ER), progesterone receptor (PR), and Human Epidermal growth factor Receptor 2 (HER2/neu) status [8].

Patients were classified according to the ASTRO consensus statement for APBI [9]. Presence or absence of lymphovascular space invasion (LVSI) was reported; however, extent was not specifically addressed. Similarly, extensive intraductal component (EIC) was reported as present without regards to size in the majority of reports. Therefore the presence of LVSI or EIC was considered cautionary. No patient was known to be BRCA1/2 positive.

Time to event and length of follow-up was calculated using the date of final surgery as day 0. An IBTR was a failure for local control (LC). LC was defined as one minus the local recurrence rate (LR). Further, IBTR were classified as "true recurrence" when they occurred in the same quadrant as the initial tumor, or "elsewhere" if they occurred in a separate quadrant. Recurrences in the supraclavicular, infraclavicular, internal mammary, intrapectoral, and axillary nodes were defined as a locoregional recurrence (LRR).

Continuous variables were compared using two-tailed *t*-tests and categorical variables were compared using Fisher's exact test with p values ≤ 0.05 considered significant. Estimates of LR, LRR, disease-free survival (DFS), cause specific survival (CSS), and overall survival (OS) were performed using the Kaplan-Meier method using SAS version 9.2 (SAS Institute Inc., Cary, NC). Survival was compared using log-rank tests. Predictive effects were analyzed using a Cox proportional hazards multivariate regression model with two-sided tests. Washington University School of Medicine Human Research Protection Office approved this study.

### Surgery

All patients underwent partial mastectomy as a part of BCT. Negative surgical margins were defined as ≥ 2 mm of tumor free tissue on all margins or removal of the breast tissue to the pectoralis fascia with no evidence of tumor invasion into the fascia. Re-excisions were often performed if the initial tumor-free margin was < 2 mm. A small number of patients who did not have either a sentinel lymph node biopsy or an axillary dissection were included if they had no evidence for axillary involvement at the time of diagnosis or treatment, this included three patients undergoing APBI for an IBTR previously treated with WBI. Axillary assessment was not required in patients with DCIS.

### Systemic therapy

Systemic therapy consisted of some combination of endocrine, cytotoxic chemotherapy, biologic therapy, or no systemic therapy. For patients that received APBI and cytotoxic chemotherapy, APBI occurred prior to cytotoxic chemotherapy in all but two cases. For patients who received WBI and cytotoxic chemotherapy, WBI occurred after cytotoxic chemotherapy.

### Interstitial implant technique

Interstitial implants (ISI) were placed using a free hand technique encompassing the surgical cavity with a 2 cm margin of breast tissue in all directions. All implants were multiplanar with an intraplane catheter spacing of 12 mm and an interplane spacing of 1.5 to 2.0 cm. The use of more than two planes was common. For the first 46 patients, ISI were placed intraoperatively with an open surgical cavity. ISI insertion via real-time ultrasound guidance (U/S) with a closed surgical cavity was predominantly used after this point.

The initial eight patients underwent two dimensional brachytherapy treatment planning using pairs of orthogonal plain films. All subsequent patients underwent three-dimensional (3D) treatment planning. Within one day of completion of ISI placement, patients underwent computed tomography (CT) simulation for 3D treatment planning. CT compatible markers were placed in each catheter. CT images were obtained using 2 mm slice thickness through the ISI volume plus several centimeters of surrounding tissue.

The CT data set was transferred to the brachytherapy planning system for selection of HDR dwell positions and optimization of their relative weights. The Plato Brachytherapy software system (Nucletron B. V., Veenendaal, The Netherlands) was used through November 2006 after which treatment planning was done using the Brachyvision system (Varian, Palo Alto, CA). The surgical cavity was identified on the CT dataset by contouring the seroma along with any surgical clips and density changes. The Planning Target Volume (PTV) was created by adding a uniform 2 cm margin to the surgical cavity contour and subsequently limited to 5 mm away from the skin surface. Pectoral muscle, chest wall, and axilla were excluded from the PTV.

Dwell positions within each catheter were separated by 5-7 mm. The dosimetric goal was to cover at least 95% of the PTV with the prescription dose while maintaining a ratio of the prescription dose to the mean central dose of ≥ 0.70. Following the report by Arthur et al. that suggested dose-volume predictors for fat necrosis [10], our dosimetric goals expanded to limit the volume receiving more than 150% (V_150_) of the prescription dose to ≤ 50 mL, V_200 _≤ 20 mL, and 1-V_150_/V_100 _≥ 0.70. Planning was accomplished by geometric optimization of the prescription dose to the mean central dose and subsequent user graphical optimization.

The prescription dose was 34 Gy in ten fractions administered twice daily with a six hour interfraction separation over five to seven days for all but two patients. One patient received 32 Gy in 8 fractions. The second patient was treated for a recurrent cancer in a previously irradiated field and received a dose of 30 Gy in 10 fractions. Treatment began one to two working days after the simulation. Catheters were removed after the last fraction.

Quality assurance was accomplished by performing an autoradiograph of the treatment plan and a manual exposure calculation that was compared to the predicted value based on the Paterson-Parker tables using the volume receiving 340 cGy [11].

### Whole breast irradiation

Patients in the WBI cohort were treated to the whole breast using tangential beams. Patients received a dose of 42.56-50.4 Gy in 1.8-2.66 Gy fractions. Most patients received a boost. 50 Gy in 200 cGy fractions to the whole breast followed by a 10 Gy boost to the tumor bed was the most frequent WBI dose prescription.

## Results

Patient characteristics are shown in Table 1. 202 patients were identified in the APBI cohort and 94 in the WBI cohort. Median follow-up exceeded 60 months for both groups. One hundred one APBI patients were ≤ 60 years (50.0%), and 37 patients were ≤ 50 years old (18.3%). Fifty-three WBI patients were ≤ 60 years old (56.4%) and 23 patients were ≤ 50 years old (24.5%). Characteristics are given for DCIS and invasive disease patients in Table 2.

**Table 1:** Patient demographics and tumor characteristics

	All Disease		
	**APBI**	**WBI**	**P value**
**Patients**	202	94	
**Months Follow-up**	64.3	64.1	0.422
**(Range)**	(2.2-96.6)	(4.4-98.4)	
**Median Age**	60.0	56.9	0.087
**(Range)**	(34.7-84.3)	(33.0-83.2)	
**Race**			< 0.001*
**Caucasian**	169 (83.7%)	52 (55.3%)	
**Black**	31 (15.4%)	42 (44.7%)	
**Other**	2 (1.0%)	0 (0%)	
**Anatomy**			
***Side***			0.803
**Right**	97 (48.0%)	47 (50.0%)	
**Left**	105 (52.0%)	47 (50.0%)	
***Quadrant***			
**UOQ**	88 (43.6%)	64 (68.1%)	< 0.001*
**UIQ**	53 (26.2%)	17 (18.1%)	
**LIQ**	29 (14.4%)	5 (5.3%)	
**LOQ**	32 (15.8%)	8 (8.5%)	
**Grade**			0.207
**DCIS**	40 (19.8%)	18 (19.1%)	
**I**	78 (38.6%)	25 (26.6%)	
**II**	55 (27.2%)	27 (28.7%)	
**III**	29 (14.4%)	20 (21.3%)	
**Unknown**	0 (0%)	4 (4.3%)	
**Stage**			
**Tis**	40 (19.8%)	18 (19.1%)	
**T1_mic_**	3 (1.5%)	4 (4.3%)	
**T1_a_**	23 (11.4%)	7 (7.4%)	
**T1_b_**	67 (33.2%)	29 (30.9%)	
**T1_c_**	54 (26.7%)	31 (33.0%)	
**T1**	147 (72.8%)	71 (75.5%)	
**T2**	15 (7.4%)	5 (5.3%)	
**Histology**			0.092
**DCIS**	40 (19.8%)	18 (19.1%)	
**Invasive Ductal**	141 (69.8%)	60 (63.8%)	
**Invasive Lobular**	8 (4.0%)	11 (11.7%)	
**Invasive Tubular**	7 (3.5%)	1 (1.1%)	
**Invasive Mucinous**	5 (2.5%)	2 (2.1%)	
**Invasive Papillary**	1 (0.5%)	2 (2.1%)	
**Estrogen Receptor**			0.464
**ER +**	167 (82.7%)	71 (71.6%)	
**ER -**	26 (12.9%)	15 (16.0%)	
**ER Unknown**	9 (4.5%)	8 (8.5%)	
**Progesterone Receptor**			0.489
**PR +**	134 (66.3%)	56 (59.6%)	
**PR -**	59 (29.2%)	30 (31.9%)	
**PR Unknown**	9 (4.5%)	8 (8.5%)	
**Her-2/Neu** **(Invasive Only)**			0.350
**Overexpressed**	14 (6.9%)	9 (4.5%)	
**Not Overexpressed**	147 (72.8%)	62 (66.0%)	
**Unknown**	41 (20.3%)	23 (24.5%)	
**Disease Type**			
**Luminal A**	128 (63.4%)	51 (54.3%)	0.353
**Luminal B**	9 (4.5%)	7 (7.5%)	
**Her-2/Neu**	5 (2.5%)	2 (2.1%)	
**Basal**	16 (7.9%)	11 (3.7%)	
**DCIS**	40 (19.8%)	18 (6.1%)	
**Unable to classify**	4 (2.0%)	5 (5.3%)	
**LVSI (Invasive Only)**			0.591
**Present**	10 (4.9%)	6 (8.1%)	
**Absent**	152 (74.5%)	70 (91.9%)	
**LCIS**			0.011*
**Present**	15 (7.4%)	17 (18.1%)	
**Absent**	187 (92.6%)	77 (81.9%)	
**EIC (Invasive Only)**			0.688
**Present**	4 (2.5%)	3 (3.9%)	
**Absent**	148 (91.4%)	73 (96.1%)	
**Unknown**	10 (6.2%)	0 (0%)	
**Endocrine Therapy** **(Invasive Only)**			0.182
**Given**	127 (78.4%)	50 (65.8%)	
**Not Given**	34 (21.0%)	21 (27.6%)	
**Unknown**	1 (0.6%)	5 (6.6%)	
**Endocrine Therapy** **(DCIS Only)**			0.756
**Given**	29 (72.5%)	14 (77.8%)	
**Not Given**	11 (27.5%)	4 (22.2%)	
**Unknown**	0 (0%)	0 (0%)	
**Cytotoxic Chemotherapy** **(Invasive Only)**			0.102
**Given**	35 (21.6%)	23 (30.3%)	
**Not Given**	127 (78.4%)	49 (64.5%)	
**Unknown**	0 (0%)	4 (5.3%)	

* Statistically significant

**Table 2:** Patient demographics and tumor characteristics for DCIS and invasive disease

	DCIS			Invasive		
	**APBI**	**WBI**	**P value**	**APBI**	**WBI**	**P value**

**Patients**	40	18		162	76	
**Months Follow-up**	69.4	72.7	0.502	62.9	62.1	0.543
**(Range)**	(13.7-92.6)	(24.6-87.6)		(2.2-96.6)	(4.4-98.4)	
**Median Age**	59.2	56.5	0.752	61.4	58.1	0.079
**(Range)**	(40.3-82.4)	(41.4-84.2)		(34.7-84.3)	(33.0-84.7)	
**Race**			0.018			< 0.001
**Caucasian**	31 (77.5%)	10 (55.6%)		138 (85.2%)	44 (57.9%)	
**Black**	9 (22.5%)	8 (44.4%)		22 (13.6%)	32 (42.1%)	
**Other**	0 (0%)	0 (0%)		2 (1.2%)	0 (0.0%)	
**Anatomy**						
***Side***			0.776			1.000
**Right**	17 (42.5%)	9 (50.0%)		80 (49.4%)	38 (50.0%)	
**Left**	23 (57.5%)	9 (50.0%)		82 (50.6%)	38 (50.0%)	
***Quadrant***			0.049			< 0.001
**UOQ**	18 (45.0%)	14 (77.8%)		70 (43.2%)	50 (65.8%)	
**UIQ**	7 (17.5%)	3 (16.7%)		46 (28.4%)	14 (18.4%)	
**LIQ**	6 (15.0%)	1 (5.6%)		23 (14.2%)	4 (5.3%)	
**LOQ**	9 (22.5%)	0 (0%)		23 (14.2%)	8 (10.5%)	
**Estrogen Receptor**			0.548			0.256
**ER +**	28 (70.0%)	12 (66.7%)		139 (85.8%)	59 (77.6%)	
**ER -**	3 (7.5%)	0 (0%)		23 (14.2%)	15 (19.7%)	
**ER Unknown**	9 (22.5%)	6 (33.3%)		0 (0%)	2 (2.6%)	
**Progesterone Receptor**			0.727			0.369
**PR +**	21 (52.5%)	9 (50.0%)		113 (69.8%)	49 (64.5%)	
**PR -**	10 (25.0%)	3 (16.7%)		49 (30.2%)	27 (35.5%)	
**PR Unknown**	9 (22.5%)	6 (33.3%)		0 (0%)	2 (2.6%)	
**LCIS**			0.084			
**Present**	1 (2.5%)	3 (16.7%)		14 (8.6%)	14 (18.4%)	0.050
**Absent**	39 (97.5%)	15 (83.3%)		148 (91.4%)	62 (81.6%)	

Seven patients treated with APBI were treated in a previously irradiated field. Three of these patients had previously received WBI as a part of BCT for a prior diagnosis of breast cancer and received repeat BCT for an IBTR diagnosed at least 10 years after the initial diagnosis. Four patients received radiation therapy for tumors treated earlier in life (two had Hodgkin's lymphoma and two had an upper extremity sarcoma).

Six patients treated via APBI did not have surgical assessment of the axilla. Of these patients, 4 had previously undergone axillary dissections so reassessment was not technically feasible. For the remaining two patients, one had a < 1 mm focus of invasive disease in the setting of LCIS and the other had a 6 mm focus of invasive tubular disease.

### APBI dosimetry

192 patients had ≥ 90% of the PTV covered by the prescription dose and 133 patients had ≥ 95% of the PTV covered by the prescription dose. Dose homogeneity as assessed by 1-V_150_/V_100 _had a median value of 0.80. Dosimetric parameter averages for the APBI patients are given in Table 3.

**Table 3:** Dosimetric parameters for multicatheter APBI treatments

	Median	Minimum	Maximum
**V_100_**	222	97.3	775
**V_150_**	43.6	19.1	190
**V_200_**	15.6	7.01	58.2
**(1-V_150_/V_100_)**	0.8	0.45	0.88
**DHI**	0.83	0.56	1.00
**PTV Volume**	154.5	57.6	552
**PTV Coverage**	148.5	56.2	520
**% PTV Coverage**	95.7%	78.5%	100%
**Cavity Volume**	18.4 cc	1.4 cc	114 cc
**Number of Catheters**	20	10	37

### Recurrence rates and survival analysis

**Figure 1:**
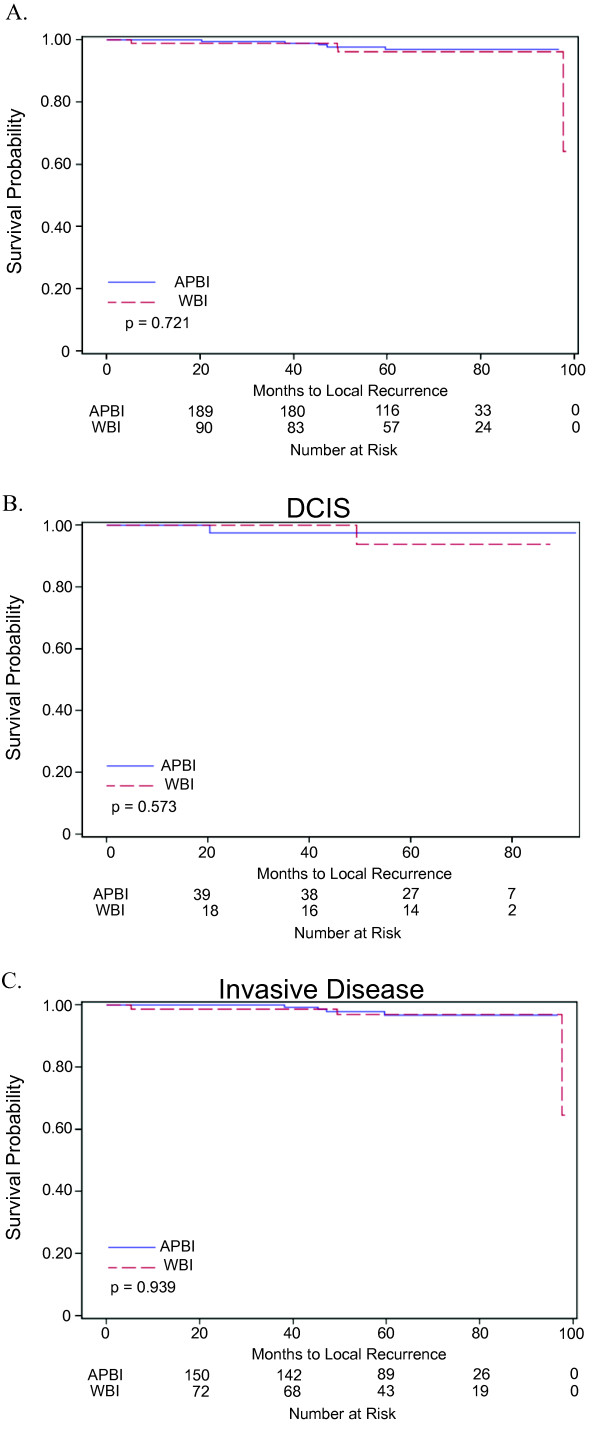
**Survival and time-to-recurrence for patients treated with WBI or APBI**. A. Survival curve demonstrating time to local recurrence for all patients stratified by type of radiation received. B. Time to local recurrence for patients treated for DCIS stratified by type of radiation received. C. Time to local recurrence for patients treated for invasive disease stratified by type of radiation received.

The LR, LRR, and DFS were similar between the groups (Table 4). Survival curves demonstrating LC for all disease, DCIS only and invasive disease only stratified by radiation method are given in Figure 1. There was no statistical difference in OS or CSS between the groups. While OS was not significantly lower, it was trending lower in the APBI group compared to the WBI cohort. A number of patients who received APBI had significant medical co-morbidities and chose APBI over WBI. These patients were opposed to omitting radiation therapy and chose APBI for the convenience of a shorter time commitment. Seven of these patients died from their pre-existing comorbidities (Table 5).

**Table 4:** 5-year survival rates and number of failures

	APBI	WBI	P value
**Overall**			
Local Recurrence Rate	3.04% (5)	3.82% (3)	0.721
Locoregional Recurrence Rate	4.25% (7)	3.82% (3)	0.902
Disease-Free Survival	94.3% (9)	93.4% (8)	0.870
Cause Specific Survival	99.4% (1)	98.9% (1)	0.954
Overall Survival	91.9% (15)	96.7% (3)	0.113
**DCIS**			
Local Recurrence Rate	2.56% (1)	6.25% (1)	0.573
Disease-Free Survival	97.4% (1)	93.8% (1)	0.573
Cause Specific Survival	100% (0)	100% (0)	-
Overall Survival	97.5% (1)	100% (1)	0.843
**Invasive Disease**			
Local Recurrence Rate	3.24% (4)	3.10% (2)	0.939
Locoregional Recurrence Rate	4.80% (6)	3.10% (2)	0.669
Disease-Free Survival	93.8% (8)	94.1% (4)	0.920
Cause Specific Survival	99.3% (1)	98.6% (1)	0.968
Overall Survival	90.4% (14)	95.6% (3)	0.093

**Table 5:** Characteristics of patients who expired and causes of death

Age at Diagnosis	APBI Class	T stage	Histology	Grade	Biomarker Pattern	Adjuvant Treatment	Radiation Modality	Time to Death (months)	Cause of Death
77.5	cautionary	2	ductal	II	+/+/-	R + E	APBI	43.76	CAD*, Anoxic Brain Injury after V Fib episode
35.6	unsuitable	2	ductal	III	-/-/+	R	APBI	60.35	Recreational Drug Overdose
45.4	unsuitable	2	ductal	II	+/+/-	R + C + E	APBI	74.68	Breast Cancer
81.8	acceptable	1a	ductal	II	+/+/-	R	APBI	2.83	Stroke/Fall
57.4	cautionary	1a	ductal	I	+/+/-	R	APBI	8.25	Cirrhosis* with Hepatocellular Cancer
51.6	cautionary	1a	ductal	III	+/-/-	R + E	APBI	35.91	Colon Cancer*
66.5	cautionary	1a	ductal	III	-/-/-	R	APBI	41.95	Myelodysplastic Syndrome
34.7	unsuitable	1b	tubular	I	+/+/-	R	APBI	2.96	Urosepsis
79.1	acceptable	1b	ductal	II	+/+/-	R + E	APBI	16.33	CAD*, MI
80.2	acceptable	1b	ductal	I	+/+/-	R	APBI	32.20	COPD*
72.3	acceptable	1b	ductal	I	+/+/-	R + E	APBI	33.71	High Grade Sarcoma*
70.7	cautionary	1b	ductal	III	-/-/-	R	APBI	46.29	Breast Cancer
50.2	cautionary	1c	ductal	III	-/-/-	R + C	APBI	5.95	Chemotherapy Toxicity
58.6	cautionary	1c	ductal	III	-/-/-	R	APBI	13.50	Ovarian Cancer*
66.1	acceptable	1c	ductal	II	+/+/-	R + E	APBI	56.48	Melanoma
68.8	acceptable	1c	ductal	II	+/+/-	R + E	APBI	58.48	CHF/COPD*
66.8	acceptable	1c	ductal	I	+/+/-	R + E	APBI	80.89	Stroke
49.1	unsuitable	Is	DCIS	II	+/+/-	R	APBI	13.70	Polycystic Kidney Disease*
67.1	cautionary	Is	DCIS	III	-/-/+	R	APBI	74.35	Pneumonia (Restrictive Lung Disease*)
70.93	acceptable	1b	ductal	I	+/+/-	R + E	WBI	14.78	M. avium infection (Chronic bronchiectasis*)
68.55	cautionary	1b	ductal	III	+/+/-	R	WBI	22.01	Breast cancer
68.76	acceptable	1c	mucinous	II	+/+/-	R + E	WBI	23.10	Parkinson's Disease
48.83	unsuitable	is	DCIS	II	+/-/-	R + E	WBI	67.02	CAD*, MI

*condition present prior to diagnosis of breast cancer; abbreviations as Table 3. CAD: coronary artery disease, COPD: chronic obstructive pulmonary disease, MI: myocardial infarction

Characteristics of patients who experienced disease recurrence are described in Table 6. In all cases with an initial diagnosis of regional or metastatic failure, no evidence of local failure was present. The patient treated via APBI who developed a regional failure at 55.0 months was found to have a nodal recurrence in a portion of the axilla that would likely have been covered using tangential beam if given WBI. The other patient treated with APBI who developed a regional recurrence was diagnosed at 17.1 months with an axillary nodal recurrence that would have been superior to the upper tangent border if treated by WBI. Both regional failures were in the APBI group and were axillary failures. Both patients had negative sentinel lymph node biopsies at initial diagnosis. It was estimated that one of the site of one of the axillary failures would have been treated with standard tangential fields if WBI had been given and the other would not have been included. All patients who developed regional or distant disease remained free of IBTR at death or last follow up.

**Table 6:** Characteristics of patients with failure

Age at Diagnosis	Menopause Status	ASTRO Category	Size	Histology	Grade	Biomarker Pattern	Node Biopsy	Adjuvant Treatment	Radiation Modality	Initial Failure	Failure (months)	Salvage Treatment	Current Status
61.7	Post	cautionary	1.00	DCIS	II	+/-/?	No	R + E	APBI	Local - E	20.3	MRM + C + E	NED
62.1	Post	acceptable	0.30	IDC	I	+/+/-	Yes	R + E	APBI	Local - T	45.3	MRM + C	NED
66.8	Post	acceptable	1.00	IDC	II	+/+/-	Yes	R + E	APBI	Local - E	59.6	SM	NED
69.3	Post	acceptable	2.00	IDC	II	+/-/-	Yes	R + E	APBI	Local - E	38.2	PM + C + WBI + E	NED
59.5	Post	cautionary	2.00	IDC	II	+/-/-	Yes	R + E + C	APBI	Local - E	47.1	MRM + C + E	NED
68.2	Post	cautionary	0.90	IDC	III	-/-/-	Yes	R	WBI	Local - E	49.5	MRM	NED
57.0	Post	cautionary	1.50	IDC	III	-/-/-	Yes	R + C	WBI	Local - T	5.2	SM	NED
56.5	Post	cautionary	0.90	DCIS	III	+/+/?	No	R	WBI	Local - T	49.3	SM	NED
62.3	Post	acceptable	0.60	IDC	II	+/+/-	Yes	R + E	WBI	Local - E	97.5	MRM + E	NED
45.4	Pre	unsuitable	3.00	IDC	II	+/+/-	Yes	R + E + C	APBI	Regional	55.0	SR + C	Expired
53.6	Post	cautionary	3.00	IDC	II	+/+/-	Yes	R + E	APBI	Regional	17.1	SR + C + R	NED
70.7	Post	cautionary	0.80	IDC	III	-/-/-	Yes	R	APBI	Metastatic	32.4	C + H	Expired
68.6	Post	cautionary	1.00	IDC	III	+/+/-	Yes	R	WBI	Metastatic	13.2	-	Expired
71.1	Post	cautionary	2.10	IDC	III	-/-/+	Yes	R + C	APBI	Metastatic	45.4	C + R	Under Treatment
56.5	Post	cautionary	2.90	IDC	III	+/+/+	Yes	R + C + E	APBI	Metastatic	60.8	R	Under Treatment
58.1	Post	cautionary	1.50	IDC	II	+/-/-	Yes	R	WBI	Metastatic	46.1	C	Under Treatment

*Abbreviations*: *SM *simple mastectomy, *MRM *modified radical mastectomy, *PM *partial mastectomy, *R *radiotherapy, *C *chemotherapy, *E *endocrine therapy, *H *Herceptin, Local-T - true recurrence, Local-E - elsewhere recurrence. Biomarker pattern shows the status for estrogen receptor, progesterone receptor, and HER2/neu using the following symbolic code: (ER)/(PR)/(HER2/neu). Presence of the biomarker is indicated by '+,' absence by '-,' unknown by '?.'

### Classification by ASTRO consensus guidelines

Using the criteria to outlined in the 2009 ASTRO consensus statement on APBI (Table 7), patients in both the WBI and APBI cohorts were categorized into one of three categories: acceptable, cautionary or unsuitable (Table 8).

**Table 7:** ASTRO APBI consensus statement categories and classification criteria

Criteria	Suitable	Cautionary	Unsuitable
**Age**	≥ 60	50-59	< 50
**Tumor Size**	≤ 2 cm	2-3 cm	> 3 cm
**T Stage**	T1	Tis and T2	T3-T4
**Nodal biopsy**	Yes	-	No
**Margins**	Negative (≥ 2 mm)	Close (< 2 mm)	Positive
**Histology**	Invasive ductal or other favorable subtypes	Invasive Lobular	-
**Pure DCIS**	No	≤ 3 cm	> 3 cm
**ER Receptor**	Positive	Negative	-
LVSI*	No	Limited/Focal	*Extensive*
EIC*	No	≤ 3 cm	*> 3 cm*

* Presence of LVSI and EIC were considered cautionary in this study. Multicentricity, multifocality, neoadjuvant chemo/endocrine therapy, and known *BRCA1/2 *mutations were not present in any patients in this study. Pathologic nodal stage was pN0 for all patients who underwent axillary assessment

**Table 8:** Distribution of patient factors across ASTRO consensus statement parameters

	Overall (%)	Age at Diagnosis	Tumor Size	T Stage	ER Status	Histology	DCIS	EIC	LVSI	Nodal Assessment
**APBI**										
**Acceptable**	58 (28.7)	101	147	147	167	194	162	158	152	156
**Cautionary**	104 (51.5)	64	15	55	35	8	40	4	10	0
**Unsuitable**	40 (19.8)	37	0	0	0	0	0	-	-	6
**WBI**										
**Acceptable**	16 (17.0)	41	71	71	71	83	76	73	70	67
**Cautionary**	47 (50.0)	30	5	23	23	11	18	3	6	0
**Unsuitable**	31 (33.0)	23	0	0	0	0	0	-	-	9

Overall, the APBI and WBI groups were significantly different with respect to ASTRO consensus category classification (p = 0.017). None of the ASTRO consensus categories predicted LR, LRR or DFS either when analyzed by treatment group or when analyzed in the entire study population. In a multivariate model for LRR in which radiation method was force entered, age, stage, radiation method, biomarker pattern, and ASTRO consensus category were all non-significant predictors.

## Discussion

Multicatheter APBI represents the first form of partial breast irradiation offered at Washington University as an alternative to WBI for a select group of early-stage breast cancer patients. Our experience suggests that this method offers similar LRR, DFS, CSS, and OS compared to WBI. Our series includes a significant number of young patients and patients treated for pure DCIS. Both of these subgroups had no significant difference in LRR, DFS, CSS or OS with APBI as compared to WBI.

The most significant limitation of this series is length of follow-up given the long natural history of breast cancer. While the low number of events is encouraging, it does limit the statistical analysis of predictors of these events. As this patient population matures, occurrences will inevitably increase, making more complex analyses possible. Selection bias may also limit the applicability of this analysis as it is a retrospective study; however, we do report a concurrent cohort of patients who were eligible for and offered APBI but who selected WBI whose outcomes were similar. It was practice in our clinic to offer each patient both options when it was felt that either option was technically feasible and appropriate for the specific patient's disease.

### Previous APBI experiences

Multicatheter APBI has been practiced for over 20 years. The Oschner clinic reported one of the first series of patients treated in this fashion. A phase I/II trial of HDR or Low Dose Rate (LDR) brachytherapy was initiated in the early 1990s to evaluate tumor control in wide-field partial breast irradiation. Patients with Tis-T2 disease less than 4 cm in diameter with 0-3 positive axillary nodes were treated. One in breast recurrence and three nodal recurrences were reported at 75 months, all three nodal recurrences in patients with extracapsular nodal disease at the time of treatment [12]. Similar early studies were also performed at other institutions and by the Radiation Therapy Oncology Group 9517 cooperative study [6,7,13,14]. An exhaustive list of APBI studies using a variety of techniques is reported in Smith et al [9].

Recent reports are congruent with our findings. McHaffee et al. report the Wisconsin experience with HDR interstitial brachytherapy using multicatheter or MammoSite balloon techniques. The majority of the 322 patients reported in the series underwent multicatheter APBI and were planned using modern 3D-CT methods. Patients received 32 Gy in 8 BID fractions or 34 Gy in 10 BID fractions. The 5-year LR was 4.8% and the 5-year DFS was 89.6% [15]. Strnad et al. reports the results from the German-Austrian Phase II trial investigating multicatheter brachytherapy in 274 patients. Inclusion criteria for this study were stricter than our study, requiring absence of LVSI and a maximum histologic grade of II/III. The 5-year and 8-year actuarial survival was reported as 97.7% and 95%, respectively for the entire cohort. DFS was 96.1% and 88% at 5 and 8 years [16]. Shah et al. reports a retrospective series of 199 patients treated via LDR or HDR interstitial brachytherapy matched to a cohort of patients treated with WBI. The LDR technique delivered 50 Gy over 96 hours at 0.52 Gy/h. The HDR technique delivered 32 Gy in 8 fractions BID or 34 Gy in 10 fractions BID. The LR rate was 5.0% for the APBI cohort and 3.8% for the matched WBI patient cohort at 12 years (ns). DFS was 91% for the APBI cohort and 87% for the WBI cohort (ns) [17]. Polgár et al. reports the Hungarian experience with APBI, the longest follow-up in the literature for the HDR multicatheter technique. Inclusion criteria included size < 2 cm, negative margins, maximum histologic grade of II/III, pN0-N1mic. Carcinoma *in situ *or lobular carcinoma were excluded. The 5- and 12-year LR was 4.4% and 9.3%, respectively. DFS was 75.3% at 12 years [18].

LR rate in 40 patients treated via APBI with pure DCIS (45% high grade) in our series was less than 3% at five years. While treatment of pure DCIS via APBI historically has been controversial, recent reports have demonstrated good local control, even when high-grade DCIS patients are included. Jeruss et al. examined outcomes of patients enrolled on the American Society of Breast Surgeons APBI MammoSite registry trial treated for pure DCIS. Eligibility criteria included size < 4.5 cm as defined mammographically, clinically negative nodes, and negative margins. One hundred ninety-four patients were identified with 36% of the patients having high-grade disease and 53% of the patients receiving endocrine therapy. Dose prescription was 34 Gy in 10 BID fractions. The 5-year LR was 3.39% and DFS was 93.2% [19]. In addition, McHaffee et al reports 32 patients treated as described above for DCIS with no recurrences at 5 years [15].

### APBI consensus statement

In 2009, ASTRO APBI consensus statement task force released guidelines for appropriate patient selection for APBI [9]. After review of the literature, three general categories were developed from a number of patient and tumor characteristics with special focus selecting patients for APBI outside of clinical trial. The GEC-ESTRO group released a similar statement in 2010 [20].

Recently, Shaitelman and colleagues reviewed patients who received APBI via the MammoSite brachytherapy technique as a part of BCT [21]. 1025 of the 1449 patients on the MammoSite Registry could be classified using the ASTRO consensus criteria. Of these patients, 419 were classified as suitable, 430 as cautionary, and 176 as unsuitable. This classification scheme did not predict different outcomes with regards to local or regional disease recurrence, DFS, CSS, or OS. The only statistically significant difference between the categories was with regards to rate of distant metastases. This suggests that the classification may predict disease that tends to be more aggressive, regardless of local control modality employed.

Beitsch and colleagues reviewed outcomes for patients treated on the MammoSite registry trial that were retrospectively classified as unsuitable by the ASTRO guidelines [22]. This analysis identified 176 patients as unsuitable and found that there was no difference in local, regional, or distant failure between the unsuitable and other classifications. In addition, only ER status was correlated with IBTR on univariate analysis when looking at all patients in the registry, suggesting that the ASTRO criteria lack power to identify a cohort of patients that are more likely to recur locally fail after APBI compared to WBI. Zauls et al. compared patients treated with WBI and APBI via MammoSite balloon therapy and did not find a difference in the time to local failure at 4-years between the treatment types for any of the three ASTRO classifications [23].

McHaffee et al. analyzed a cohort of 322 patients who received APBI via multicatheter brachytherapy or MammoSite balloon brachytherapy and retrospectively classified patients using the ASTRO guidelines. ASTRO classification criteria including margin status, EIC, histology and size, but not age did predict decreased IBTR and LRR. Overall, the reported 5-year IBTR rate for patients classified as suitable was 1.6% compared to 6.6% for patients in the unsuitable cohort [15].

While APBI has been successfully adopted as treatment modality by many institutions, there is limited phase III data available guide patient selection. Two large, multi-institutional phase III trials, NSABP B-39/RTOG 0413 and the GEC-ESTRO APBI trial are underway will likely be the sources for the identification criteria that will discriminate between patients that will or will not be adversely affected with regards to disease control based on treatment technique, if any exist. These studies include patients with high-risk disease characteristics and also allow for variation in APBI technique. While these studies will help clarify outcomes and identify sub-populations of higher-risk patients not appropriate for APBI, these data will likely not be available for multiple years. In the interim this report offers additional evidence regarding the safety and efficacy of APBI.

## Competing interests

The authors declare that they have no competing interests.

## Authors' contributions

DF participated in the study design, collected and analyzed patient data, and drafted the manuscript. AG participated in the study design and helped draft the manuscript. TD performed the statistical analyses for the study. JM interpreted data and helped draft the manuscript. MN interpreted data and helped draft the manuscript. RA interpreted data and helped draft the manuscript. WE interpreted data and helped draft the manuscript. TE interpreted data and helped draft the manuscript. MM helped collect patient data and helped draft the manuscript. IZ participated in its design and coordination. All authors read and approved the final manuscript.

## References

[B1] Fisher B Jeong J-H Anderson S Bryant J Fisher ER Wolmark N Twenty-five-year follow-up of a randomized trial comparing radical mastectomy, total mastectomy, and total mastectomy followed by irradiation New Engl J Med 2002 347 567 575 10.1056/NEJMoa020128 12192016

[B2] Veronesi U Cascinelli N Mariani L Greco M Saccozzi R Luini A Aguilar M Marubini E Twenty-year follow-up of a randomized study comparing breast-conserving surgery with radical mastectomy for early breast cancer New Engl J Med 2002 347 1227 1232 10.1056/NEJMoa020989 12393819

[B3] Fisher B Dignam J Wolmark N Mamounas E Costantino J Poller W Fisher ER Wickerham DL Deutsch M Margolese R Lumpectomy and radiation therapy for the treatment of intraductal breast cancer: findings from National Surgical Adjuvant Breast and Bowel Project B-17 J Clin Oncol 1998 16 441 452 9469327 10.1200/JCO.1998.16.2.441

[B4] Julien JP Bijker N Fentiman IS Peterse JL Delledonne V Rouanet P Avril A Sylvester R Mignolet F Bartelink H Van Dongen JA Radiotherapy in breast-conserving treatment for ductal carcinoma in situ: first results of the EORTC randomised phase III trial 10853 Lancet 2000 355 528 533 10.1016/S0140-6736(99)06341-2 10683002

[B5] Holmberg L Garmo H Granstrand B Ringberg A Arnesson L-G Sandelin K Karlsson P Anderson H Emdin S Absolute risk reductions for local recurrence after postoperative radiotherapy after sector resection for ductal carcinoma in situ of the breast J Clin Oncol 2008 26 1247 1252 10.1200/JCO.2007.12.7969 18250350

[B6] Polgár C Fodor J Major T Németh G Lövey K Orosz Z Sulyok Z Takácsi-Nagy Z Kásler M Breast-conserving treatment with partial or whole breast irradiation for low-risk invasive breast carcinoma-5-year results of a randomized trial Int J Radiat Oncol Biol Phys 2007 69 694 702 10.1016/j.ijrobp.2007.04.022 17531400

[B7] Kuske RR Winter K Arthur DW Bolton J Rabinovitch R White J Hanson W Wilenzick RM Phase II trial of brachytherapy alone after lumpectomy for select breast cancer: toxicity analysis of RTOG 95-17 Int J Radiat Oncol Biol Phys 2006 65 45 51 10.1016/j.ijrobp.2005.11.027 16503383

[B8] Nguyen PL Taghian AG Katz MS Niemierko A Abi Raad RF Boon WL Bellon JR Wong JS Smith BL Harris JR Breast cancer subtype approximated by estrogen receptor, progesterone receptor, and HER-2 is associated with local and distant recurrence after breast-conserving therapy J Clin Oncol 2008 26 2373 2378 10.1200/JCO.2007.14.4287 18413639

[B9] Smith BD Arthur DW Buchholz TA Haffty BG Hahn CA Hardenbergh PH Julian TB Marks LB Todor DA Vicini FA Accelerated partial breast irradiation consensus statement from the American Society for Radiation Oncology (ASTRO) Int J Radiat Oncol Biol Phys 2009 74 987 1001 10.1016/j.ijrobp.2009.02.031 19545784

[B10] Arthur DW Wazer DE Koo D Shah N Berle L Cuttino L Yunes M Rivard M Todor D Tong S The importance of dose volume histogram evaluation in partial breast brachytherapy: a study of dosimetric parameters Int J Radiat Oncol Biol Phys 2003 57 S361 S362

[B11] Williamson JF Brenner DJ Halperin E, Perez CA, Brady LW Physics and Biology of Brachytherapy Principles and Practice of Radiation Oncology 2008 5 Lippincott Williams & Wilkins

[B12] King TA Bolton JS Kuske RR Fuhrman GM Scroggins TG Jiang XZ Long-term results of wide-field brachytherapy as the sole method of radiation therapy after segmental mastectomy for T(is,1,2) breast cancer Am J Surg 2000 180 299 304 10.1016/S0002-9610(00)00454-2 11113440

[B13] Wazer DE Berle L Graham R Chung M Rothschild J Graves T Cady B Ulin K Ruthazer R DiPetrillo TA Preliminary results of a phase I/II study of HDR brachytherapy alone for T1/T2 breast cancer Int J Radiat Oncol Biol Phys 2002 53 889 897 10.1016/S0360-3016(02)02824-9 12095554

[B14] Vicini FA Baglan KL Kestin LL Mitchell C Chen PY Frazier RC Edmundson G Goldstein NS Benitez P Huang RR Martinez A Accelerated treatment of breast cancer J Clin Oncol 2001 19 1993 2001 11283132 10.1200/JCO.2001.19.7.1993

[B15] McHaffie DR Patel RR Adkison JB Das RK Geye HM Cannon GM Outcomes after accelerated partial breast irradiation in patients with ASTRO consensus statement cautionary features Int J Radiat Oncol Biol Phys 2011 81 46 51 10.1016/j.ijrobp.2010.05.011 20732760

[B16] Strnad V Hildebrandt G Pötter R Hammer J Hindemith M Resch A Spiegl K Lotter M Uter W Bani M Accelerated partial breast irradiation: 5-year results of the german-austrian multicenter phase ii trial using interstitial multicatheter brachytherapy alone after breast-conserving surgery Int J Radiat Oncol Biol Phys 2011 80 17 24 10.1016/j.ijrobp.2010.01.020 20605365

[B17] Shah C Antonucci JV Wilkinson JB Wallace M Ghilezan M Chen P Lewis K Mitchell C Vicini F Twelve-year clinical outcomes and patterns of failure with accelerated partial breast irradiation versus whole-breast irradiation: results of a matched-pair analysis Radiother Oncol 2011 110 210 214 21497927 10.1016/j.radonc.2011.03.011

[B18] Polgár C Major T Fodor J Sulyok Z Somogyi A Lövey K Németh G Kásler M Accelerated partial-breast irradiation using high-dose-rate interstitial brachytherapy: 12-year update of a prospective clinical study Radiother Oncol 2010 94 274 279 10.1016/j.radonc.2010.01.019 20181401

[B19] Jeruss JS Kuerer HM Beitsch PD Vicini FA Keisch M Update on DCIS outcomes from the American Society of Breast Surgeons accelerated partial breast irradiation registry trial Ann Surg Oncol 2011 18 65 71 10.1245/s10434-010-1192-z 20577822 PMC3019276

[B20] Polgar C Van Limbergen E Potter R Kovacs G Polo A Lyczek J Hildebrandt G Niehoff P Guinot JL Guedea F Patient selection for accelerated partial-breast irradiation (APBI) after breast-conserving surgery: recommendations of the Groupe Europeen de Curietherapie-European Society for Therapeutic Radiology and Oncology (GEC-ESTRO) breast cancer working group based on clinical evidence (2009) Radiother Oncol 2010 94 264 273 10.1016/j.radonc.2010.01.014 20181402

[B21] Shaitelman SF Vicini FA Beitsch P Haffty B Keisch M Lyden M Five-year outcome of patients classified using the American Society for Radiation Oncology consensus statement guidelines for the application of accelerated partial breast irradiation: an analysis of patients treated on the American Society of Breast Surgeons MammoSite Registry Trial Cancer 2010 116 4677 4685 10.1002/cncr.25383 20602483

[B22] Beitsch P Vicini F Keisch M Haffty B Shaitelman S Lyden M Five-year outcome of patients classified in the "unsuitable" category using the American Society of Therapeutic Radiology and Oncology (ASTRO) Consensus Panel guidelines for the application of accelerated partial breast irradiation: an analysis of patients treated on the American Society of Breast Surgeons MammoSite^® ^Registry trial Ann Surg Oncol 2010 17 Suppl 3 219 225 20853036 10.1245/s10434-010-1231-9

[B23] Zauls AJ Watkins JM Wahlquist AE Brackett NC Aguero EG Baker MK Jenrette JM Garrett-Mayer E Harper JL Outcomes in Women Treated with MammoSite Brachytherapy or Whole Breast Irradiation Stratified by ASTRO Accelerated Partial Breast Irradiation Consensus Statement Groups Int J Radiat Oncol Biol Phys 2010 in press 10.1016/j.ijrobp.2010.08.034 20951508

